# Supplementary material for: Structural phase transition, s±-wave pairing, and magnetic stripe order in bilayered superconductor La3Ni2O7 under pressure
Source: Nat Commun. 2024 Mar 19;15:2470. doi: 10.1038/s41467-024-46622-z (PMC10951331; doi:10.1038/s41467-024-46622-z)
Supplement: Supplementary file 1 — Supplementary Information [file 41467_2024_46622_MOESM1_ESM.pdf]

Supplementary Information

Structural phase transition, $s_\pm$-wave pairing, and magnetic stripe order in bilayered superconductor $La_3Ni_2O_7$ under pressure

Yang Zhang,[1] Ling-Fang Lin,[1,*] Adriana Moreo,[1,2] Thomas A. Maier,[3] and Elbio Dagotto[1,2]

[1]*Department of Physics and Astronomy, University of Tennessee, Knoxville, Tennessee 37996, USA*
[2]*Materials Science and Technology Division, Oak Ridge National Laboratory, Oak Ridge, Tennessee 37831, USA*
[3]*Computational Sciences and Engineering Division, Oak Ridge National Laboratory, Oak Ridge, Tennessee 37831, USA*

I. SUPPLEMENTARY NOTE I: PHONON SPECTRUM UNDER PRESSURE

We calculated the phonon spectrum of the Fmmm and Amam phases of $La_3Ni_2O_7$ (LNO) for different pressures, by using the density functional perturbation theory approach [1–3] analyzed by the PHONONPY software in the primitive unit cell [4, 5]. Below 10.5 GPa, the phonon dispersion spectra clearly display imaginary frequencies at high symmetry points for the Fmmm structure of LNO. This same Fmmm phase becomes stable without any imaginary frequency from 10.6 GPa to 50 GPa, the maximum value we studied, as shown in Supplementary Fig. 1 and Supplementary Fig. 2. For the Amam phase of LNO, there is no imaginary frequency obtained in the phonon dispersion spectra, indicating that the Amam phase is stable from 0 to 14 GPa (see Supplementary Fig. 3). To avoid repeating too many displays, we only show the phonon spectrum of several key values of pressures for Fmmm and Amam phases.

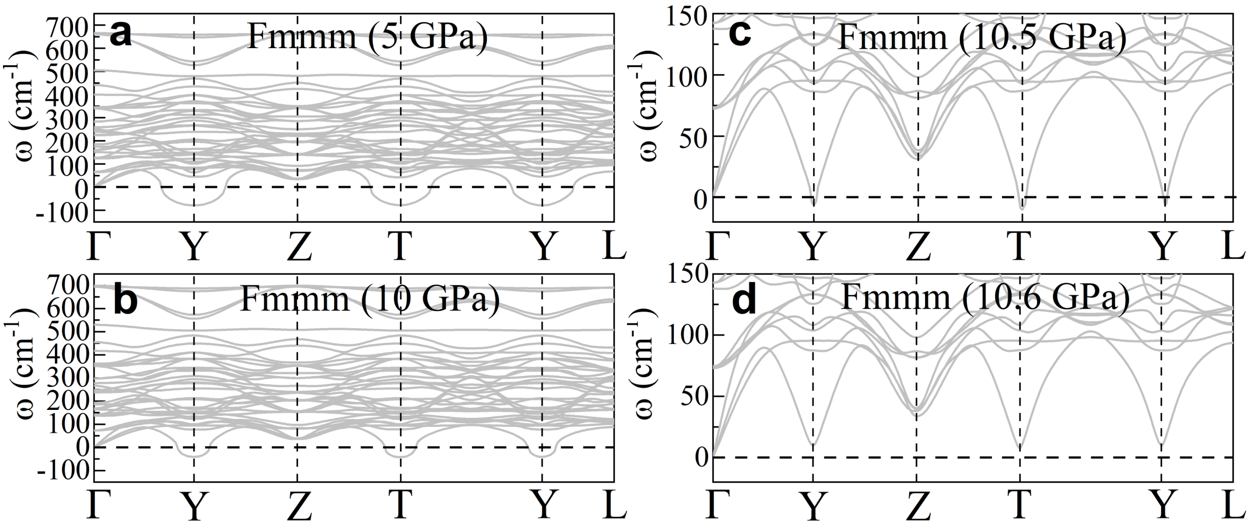

Supplementary Figure 1: **Phonon spectrum of the Fmmm phase under pressure.** Phonon spectrum of LNO for the Fmmm (No. 69) phase at **a** 5 GPa, **b** 10 GPa, **c** 10.5 GPa, and **d** 10.6 GPa, respectively. For the Fmmm phase, the coordinates of the high-symmetry points in the Brillouin zone (BZ) are Γ = (0, 0, 0), Y = (0.5, 0, 0.5), Z = (0.5, 0.5, 0), T = (0, 0.5, 0.5), and L = (0.5, 0.5, 0.5).

*Electronic address: lflin@utk.edu

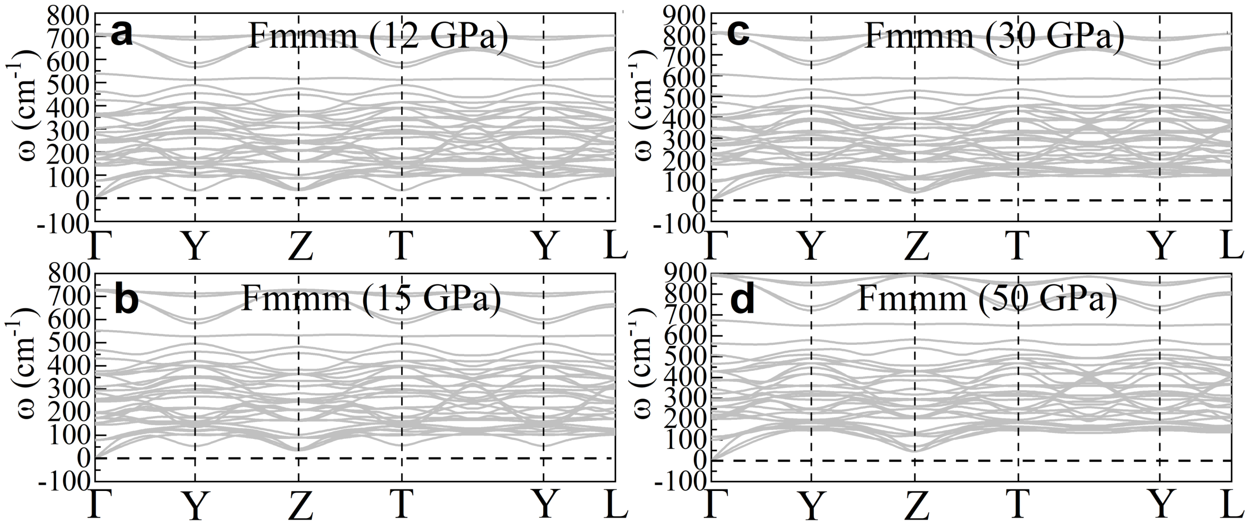

Supplementary Figure 2: **Phonon spectrum of the Fmmm phase under pressure.** Phonon spectrum of LNO for the Fmmm (No. 69) phase at **a** 12 GPa, **b** 15 GPa, **c** 30 GPa, and **d** 50 GPa, respectively. For the Fmmm phase, the coordinates of the high-symmetry points in the BZ are Γ = (0, 0, 0), Y = (0.5, 0, 0.5), Z = (0.5, 0.5, 0), T = (0, 0.5, 0.5), and L = (0.5, 0.5, 0.5).

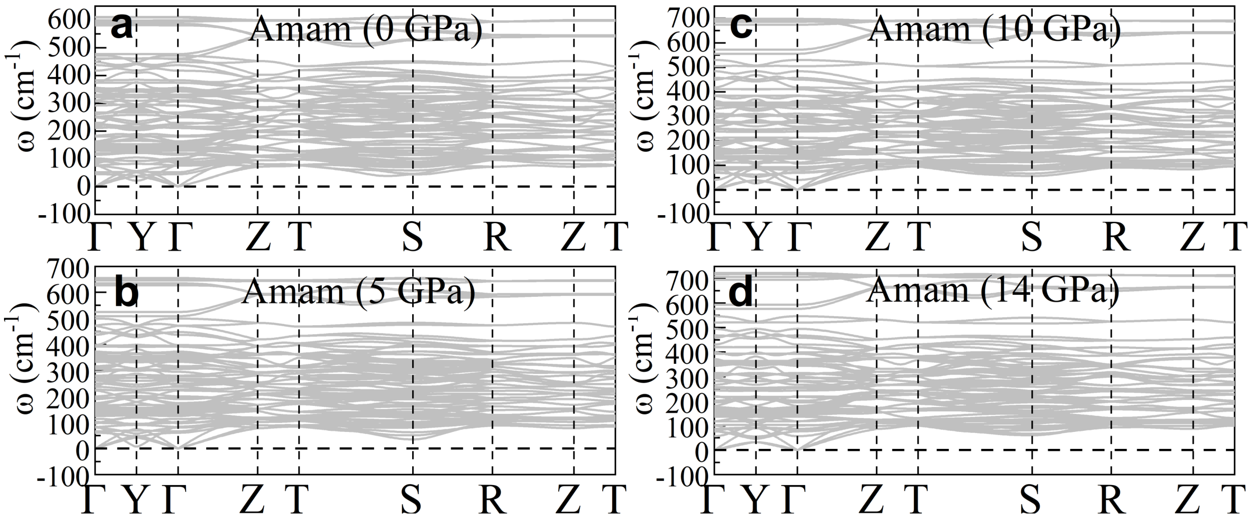

Supplementary Figure 3: **Phonon spectrum of the Amam phase under pressure.** Phonon spectrum of LNO for the Amam (No. 63) phase at **a** 0 GPa, **b** 5 GPa, **c** 10 GPa, and **d** 14 GPa, respectively. For the Amam phase, the coordinates of the high-symmetry points in the BZ are Γ = (0, 0, 0), Y = (0.5, 0.5, 0), Z = (0, 0, 0.5), T = (0.5, 0.5, 0.5), S = (0, 0.5, 0) and R = (0, 0.5, 0.5).

II. SUPPLEMENTARY NOTE II: HOPPINGS AND FERMI SURFACE UNDER PRESSURE

Based on the hoppings and crystal-field splitting obtained from the maximally localized Wannier functions [6], we calculated the Fermi surfaces for different pressures (see Supplementary Fig. 4), by using a bilayer four-band e_g-orbital tight binding (TB) model with nearest-neighbor (NN) hopping (see Supplementary Fig. 5). The Fermi level is made of two pockets (α and β) with a mixture of $d_{3z^2-r^2}$ and $d_{x^2-y^2}$ orbitals, while the γ pocket is made almost exclusively of the $d_{3z^2-r^2}$ orbital.

For the comparison, the calculated Density-functional theory (DFT) Fermi surfaces of the Fmmm phase under pressure are also shown in Supplementary Fig. 6. The main characters of those Fermi surfaces, namely hole pocket γ and two electron sheets (α and β) are qualitatively in agreement with the present DFT and TB calculations.

Below is the list of hopping amplitudes for the Fmmm phase at several pressures, deduced from DFT bands and

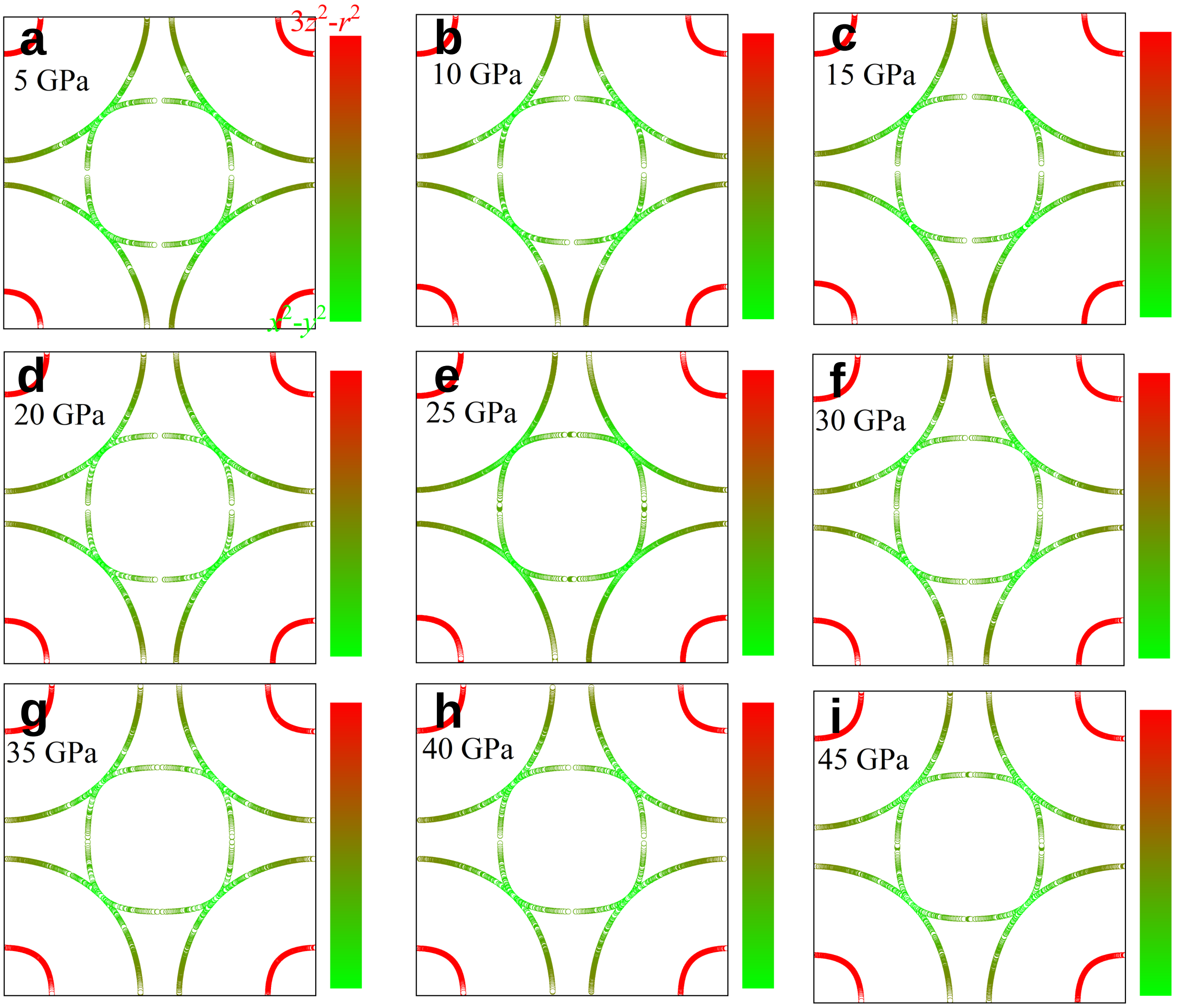

Supplementary Figure 4: **Evolution of the Fermi surface under pressure in the Fmmm phase. a-i** Fermi surfaces from 5 to 45 GPa obtained from TB calculations. Here, the four-band e_g-orbital TB model was used, with NN hoppings in a bilayer lattice for the overall filling $n = 3$ (1.5 electrons per site). Red and green represent the contribution from $d_{3z^2-r^2}$ and $d_{x^2-y^2}$ orbitals, respectively. **a** 5 GPa, **b** 10 GPa, **c** 15 GPa, **d** 20 GPa, **e** 25 GPa, **f** 30 GPa, **g** 35 GPa, **h** 40 GPa, and **i** 45 GPa, respectively.

the fitting near the Fermi level using a tight-binding two-orbital model. The values at 0 GPa and 50 GPa can be found in the main text. Varying pressure, the hoppings only weakly change.

A. Hoppings at 5 GPa

$$t_{\vec{x}} = \begin{bmatrix} d_{z^2} & d_{x^2-y^2} \\ -0.094 & 0.216 \\ 0.216 & -0.468 \end{bmatrix}, \quad \text{(Supplementary Equation1)}$$

$$t_{\vec{y}} = \begin{bmatrix} d_{z^2} & d_{x^2-y^2} \\ -0.094 & -0.216 \\ -0.216 & -0.468 \end{bmatrix}, \quad \text{(Supplementary Equation2)}$$

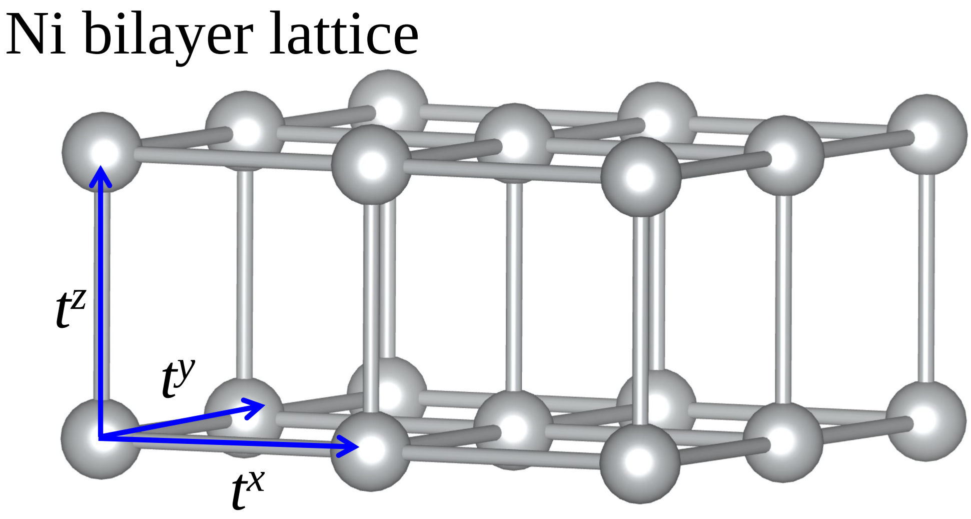

Supplementary Figure 5: **Sketch of the Ni bilayer lattice.** Sketch of the Ni bilayer lattice structure used in the TB model with NN hopping.

$$t_{\vec{z}} = \begin{bmatrix} d_{z^2} & d_{x^2-y^2} \\ -0.611 & 0.000 \\ 0.000 & 0.000 \end{bmatrix}. \quad \text{(Supplementary Equation3)}$$

B. Hoppings at 10 GPa

$$t_{\vec{x}} = \begin{bmatrix} d_{z^2} & d_{x^2-y^2} \\ -0.097 & 0.224 \\ 0.224 & -0.480 \end{bmatrix}, \quad \text{(Supplementary Equation4)}$$

$$t_{\vec{y}} = \begin{bmatrix} d_{z^2} & d_{x^2-y^2} \\ -0.097 & -0.224 \\ -0.224 & -0.480 \end{bmatrix}, \quad \text{(Supplementary Equation5)}$$

$$t_{\vec{z}} = \begin{bmatrix} d_{z^2} & d_{x^2-y^2} \\ -0.625 & 0.000 \\ 0.000 & 0.000 \end{bmatrix}. \quad \text{(Supplementary Equation6)}$$

C. Hoppings at 15 GPa

$$t_{\vec{x}} = \begin{bmatrix} d_{z^2} & d_{x^2-y^2} \\ -0.100 & 0.230 \\ 0.230 & -0.490 \end{bmatrix}, \quad \text{(Supplementary Equation7)}$$

$$t_{\vec{y}} = \begin{bmatrix} d_{z^2} & d_{x^2-y^2} \\ -0.100 & -0.230 \\ -0.230 & -0.490 \end{bmatrix}, \quad \text{(Supplementary Equation8)}$$

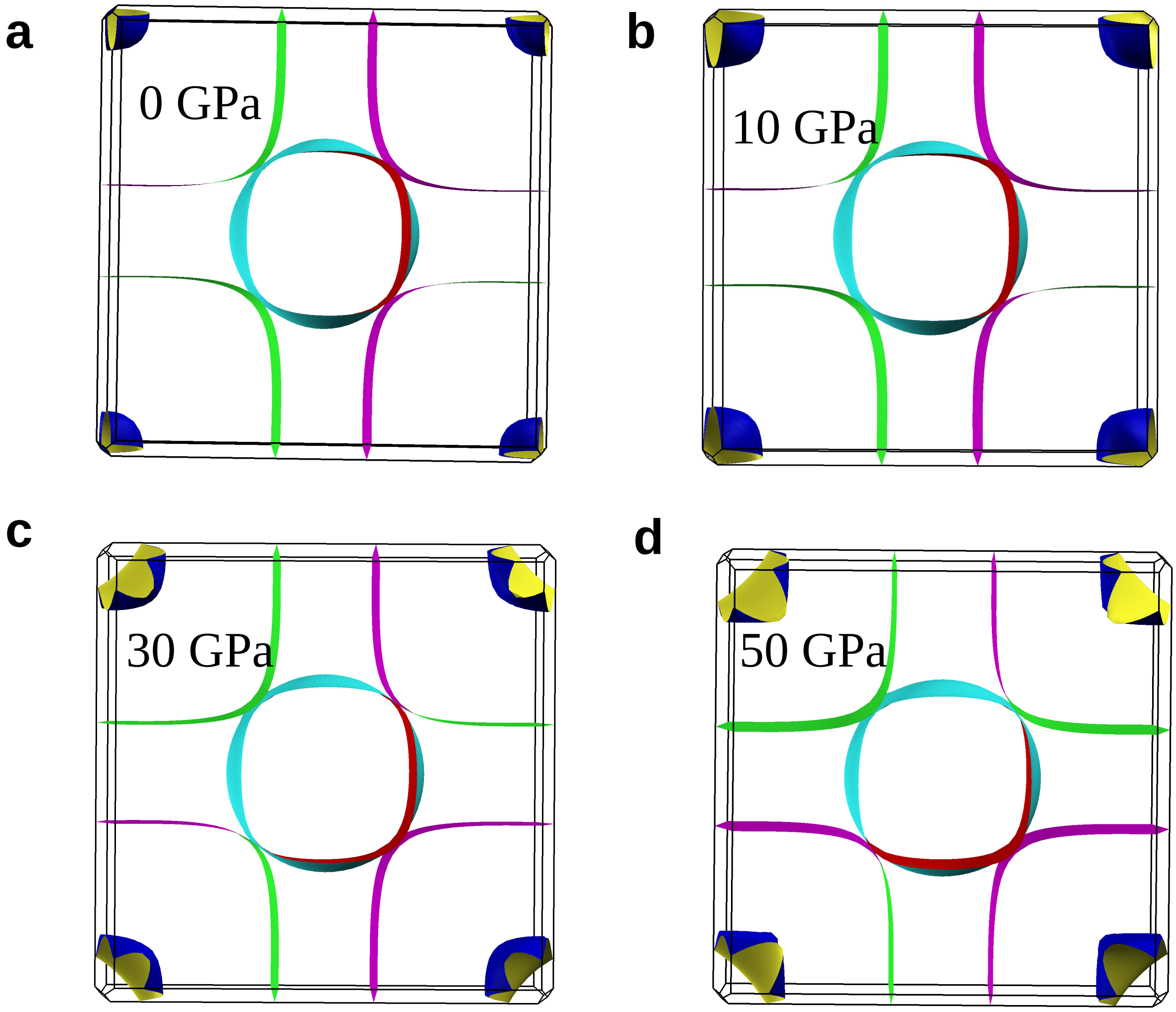

Supplementary Figure 6: **Evolution of the calculated DFT Fermi surface under pressure in the Fmmm phase. a** 0 GPa, **b** 10 GPa, **c** 30 GPa, and **d** 50 GPa, respectively.

$$t_{\vec{z}} = \begin{bmatrix} d_{z^2} & d_{x^2-y^2} \\ -0.640 & 0.000 \\ 0.000 & 0.000 \end{bmatrix}. \qquad \text{(Supplementary Equation9)}$$

D. Hoppings at 20 GPa

$$t_{\vec{x}} = \begin{bmatrix} d_{z^2} & d_{x^2-y^2} \\ -0.106 & 0.238 \\ 0.238 & -0.505 \end{bmatrix}, \qquad \text{(Supplementary Equation10)}$$

$$t_{\vec{y}} = \begin{bmatrix} d_{z^2} & d_{x^2-y^2} \\ -0.106 & -0.238 \\ -0.238 & -0.505 \end{bmatrix}, \qquad \text{(Supplementary Equation11)}$$

$$t_{\vec{z}} = \begin{bmatrix} d_{z^2} & d_{x^2-y^2} \\ -0.654 & 0.000 \\ 0.000 & 0.000 \end{bmatrix}. \qquad \text{(Supplementary Equation12)}$$

E. Hoppings at 25 GPa

$$t_{\vec{x}} = \begin{bmatrix} d_{z^2} & d_{x^2-y^2} \\ -0.110 & 0.243 \\ 0.243 & -0.515 \end{bmatrix}, \qquad \text{(Supplementary Equation13)}$$

$$t_{\vec{y}} = \begin{bmatrix} d_{z^2} & d_{x^2-y^2} \\ -0.110 & -0.243 \\ -0.243 & -0.515 \end{bmatrix}, \qquad \text{(Supplementary Equation14)}$$

$$t_{\vec{z}} = \begin{bmatrix} d_{z^2} & d_{x^2-y^2} \\ -0.666 & 0.000 \\ 0.000 & 0.000 \end{bmatrix}. \qquad \text{(Supplementary Equation15)}$$

F. Hoppings at 30 GPa

$$t_{\vec{x}} = \begin{bmatrix} d_{z^2} & d_{x^2-y^2} \\ -0.113 & 0.250 \\ 0.250 & -0.526 \end{bmatrix}, \qquad \text{(Supplementary Equation16)}$$

$$t_{\vec{y}} = \begin{bmatrix} d_{z^2} & d_{x^2-y^2} \\ -0.113 & -0.250 \\ -0.250 & -0.526 \end{bmatrix}, \qquad \text{(Supplementary Equation17)}$$

$$t_{\vec{z}} = \begin{bmatrix} d_{z^2} & d_{x^2-y^2} \\ -0.676 & 0.000 \\ 0.000 & 0.000 \end{bmatrix}. \qquad \text{(Supplementary Equation18)}$$

G. Hoppings at 35 GPa

$$t_{\vec{x}} = \begin{bmatrix} d_{z^2} & d_{x^2-y^2} \\ -0.116 & 0.256 \\ 0.256 & -0.534 \end{bmatrix}, \qquad \text{(Supplementary Equation19)}$$

$$t_{\vec{y}} = \begin{bmatrix} d_{z^2} & d_{x^2-y^2} \\ -0.116 & -0.256 \\ -0.256 & -0.534 \end{bmatrix}, \qquad \text{(Supplementary Equation20)}$$

$$t_{\vec{z}} = \begin{bmatrix} d_{z^2} & d_{x^2-y^2} \\ -0.686 & 0.000 \\ 0.000 & 0.000 \end{bmatrix}. \qquad \text{(Supplementary Equation21)}$$

H. Hoppings at 40 GPa

$$t_{\vec{x}} = \begin{bmatrix} d_{z^2} & d_{x^2-y^2} \\ -0.118 & 0.260 \\ 0.260 & -0.540 \end{bmatrix}, \quad \text{(Supplementary Equation22)}$$

$$t_{\vec{y}} = \begin{bmatrix} d_{z^2} & d_{x^2-y^2} \\ -0.118 & -0.260 \\ -0.260 & -0.540 \end{bmatrix}, \quad \text{(Supplementary Equation23)}$$

$$t_{\vec{z}} = \begin{bmatrix} d_{z^2} & d_{x^2-y^2} \\ -0.696 & 0.000 \\ 0.000 & 0.000 \end{bmatrix}. \quad \text{(Supplementary Equation24)}$$

I. Hoppings at 45 GPa

$$t_{\vec{x}} = \begin{bmatrix} d_{z^2} & d_{x^2-y^2} \\ -0.121 & 0.263 \\ 0.263 & -0.546 \end{bmatrix}, \quad \text{(Supplementary Equation25)}$$

$$t_{\vec{y}} = \begin{bmatrix} d_{z^2} & d_{x^2-y^2} \\ -0.121 & -0.263 \\ -0.263 & -0.546 \end{bmatrix}, \quad \text{(Supplementary Equation26)}$$

$$t_{\vec{z}} = \begin{bmatrix} d_{z^2} & d_{x^2-y^2} \\ -0.706 & 0.000 \\ 0.000 & 0.000 \end{bmatrix}. \quad \text{(Supplementary Equation27)}$$

III. SUPPLEMENTARY NOTE III: ADDITIONAL DFT MAGNETIC STATES RESULTS

Here, we considered three possible in-plane spin orders: A-AFM with wavector (0,0), G-AFM with (π, π), and stripe with (π, 0) orders in the plane. The magnetic coupling between layers was considered to be antiferromagnetic (AFM) in all cases. We used the optimized crystal structures at different pressures. Then, the differences in total energy and enthalpy between different magnetic configurations are the same due to having the same crystal structures at different pressures.

As shown in Supplementary Fig. 7, by *decreasing* the Hund coupling in units of U as compared with values used in the main text, namely by using $J = 0.4$ eV and $U = 4$ eV, the G-type AFM order now becomes the lowest energy state as pressure increases instead of the stripe magnetic order, due to the enhanced AFM Heisenberg interaction induced by the larger intraorbital hopping under pressure. This highlights that the stripe order, and associated $s^{\pm}$ pairing, is stable when the Hund coupling is robust.

The calculated magnetic moments of different magnetic phases all decrease when increasing pressure, as shown in Supplementary Fig. 8.

In addition, we also showed the band structures of the magnetic stripe phase from $J = 1$ eV to $J = 0.4$ eV at 25 GPa, where U is considered to be 4 eV, as displayed in Supplementary Fig. 9.

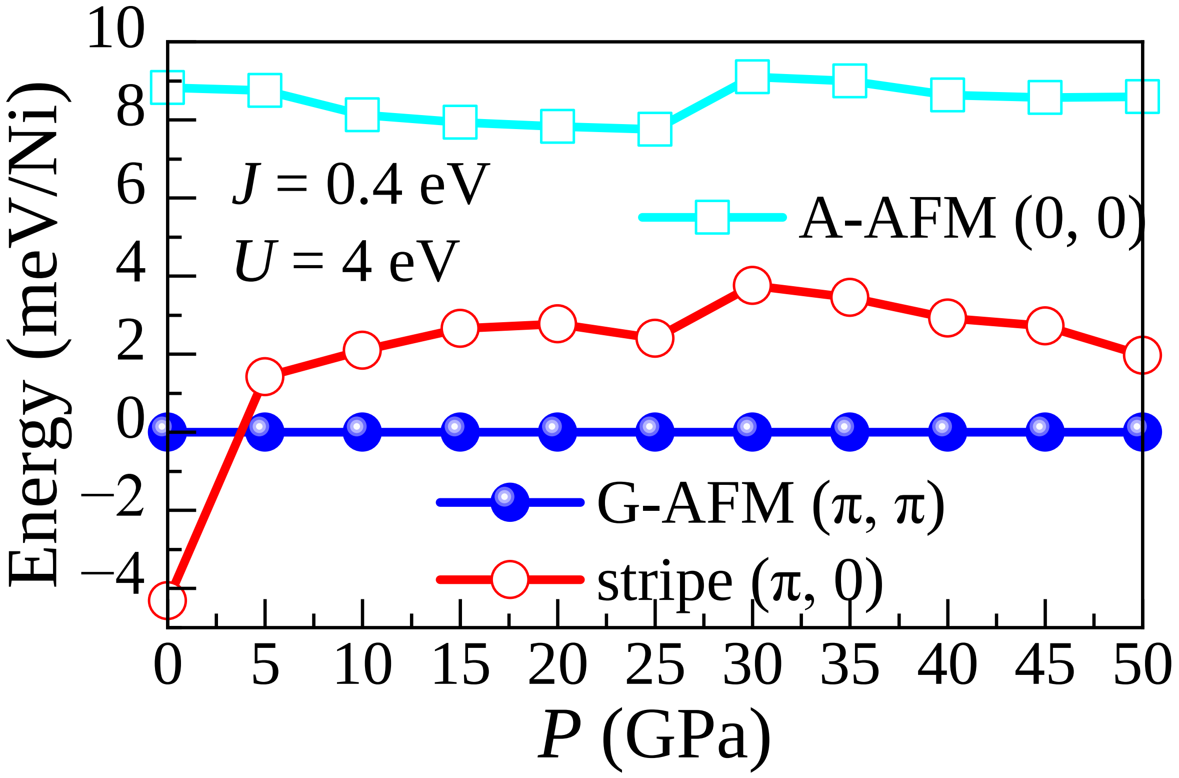

Supplementary Figure 7: **Magnetic results at $J = 0.4$ eV.** The DFT calculated energies for $J = 0.4$ eV corresponding to different magnetic configurations vs different values of pressure, at $U = 4$ eV.

IV. SUPPLEMENTARY NOTE IV: CONTRIBUTION OF THE SUSCEPTIBILITY

In Fig. 5 of the main text, we calculated the random phase approximation (RPA) susceptibility. Here, we briefly discuss the contribution of the intraorbital and interorbital components. For our RPA study, the susceptibility in Fig. 5 of the main text is renormalized and all the contributions are mixed due to the matrix form of the RPA equations. To better understand which contributions are dominant, we look at 0 GPa, where this is the bare, i.e., unrenormalized susceptibility. Below are the results for the orbital contributions at 0 GPa.

As shown in Supplementary Fig. 10, χ_0 has two peaks, one close to (π, 0) and another one between (π, 0) and (π, π). Taking into account electronic interactions through the RPA will strongly enhance the (π, 0) peak but not the other one. In addition, we also plotted the dominant contributions of the three orbitals (see the blue, red, and green curves). Adding them up gives a result very close to the black curve. Here, those orbitals are indexed as "0" for the $d_{x^2-y^2}$ orbital in layer1,"1" for the $d_{3z^2-r^2}$ orbital in layer1, "2" for the $d_{x^2-y^2}$ orbital in layer2, and "3" for the $d_{3z^2-r^2}$ orbital in layer2. Clearly, the scattering between the $d_{3z^2-r^2}$ orbitals dominates. The inter-orbital scattering between the layers (1133) is almost as strong as the intra-orbital $d_{3z^2-r^2}$ scattering within a layer.

In addition, we also show the diagonal and off-diagonal contributions to the RPA spin susceptibility, as displayed in Supplementary Fig. 11. Here, the off-diagonal contribution to the peak around (π, 0) is just as large as the diagonal one due to the $d_{3z^2-r^2}$ scattering between the two layers.

V. SUPPLEMENTARY NOTE V: IMPORTANCE OF M POINT POCKET

To better understand the importance of the γ pocket in the pairing process, we calculated the Fermi surface by changing Δ to 0.6 eV, while other parameters remain the same as for 0 GPa. The hole band sinks below the Fermi level and the γ-pocket disappears, as shown in Supplementary Fig. 12. Then, the most notorious aspect to notice is that the pockets at (π, π), and rotated equivalent points, are absent. This goes together with the suppression of $s^{\pm}$ pairing.

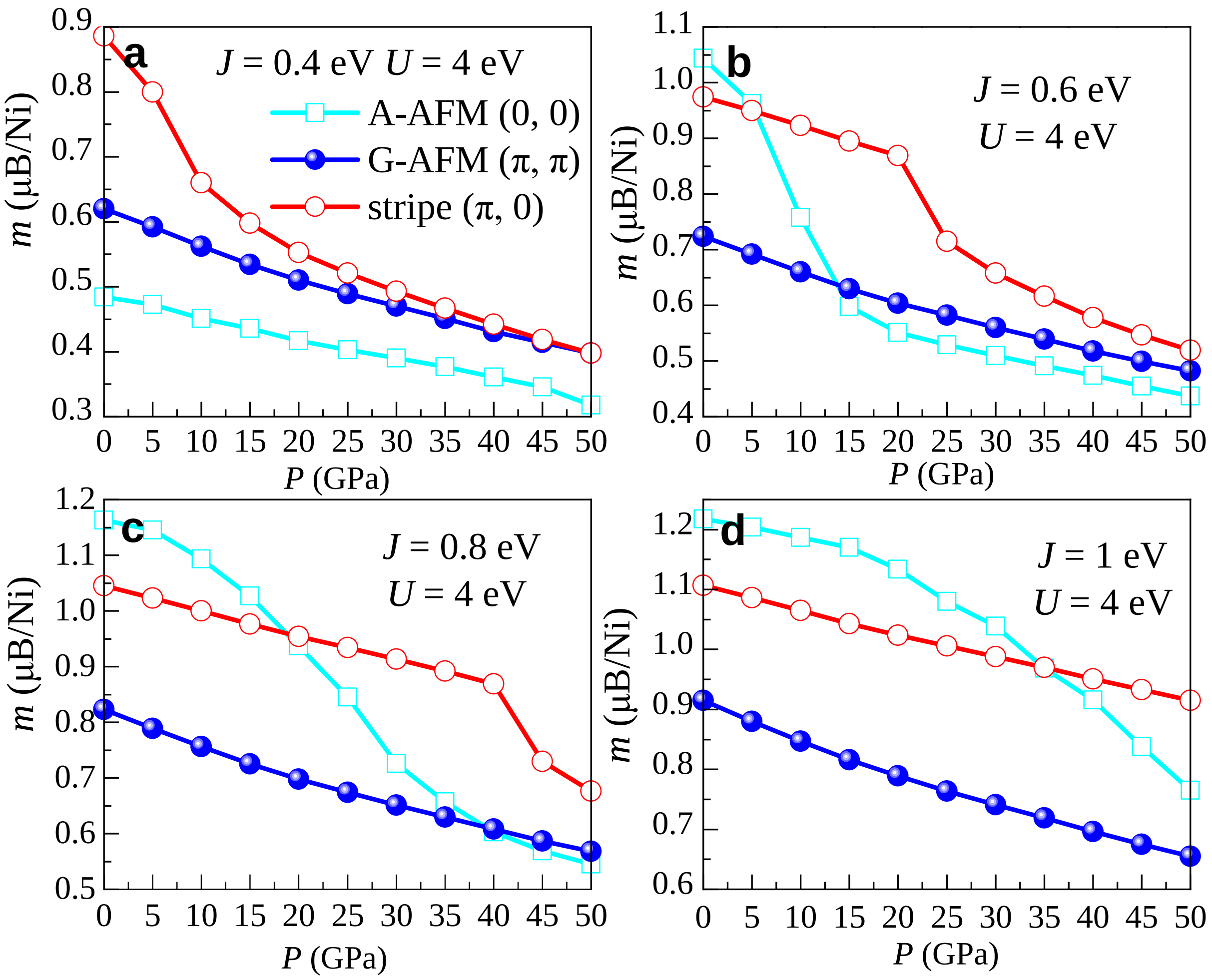

Supplementary Figure 8: **The calculated magnetic moments under pressure.** The DFT calculated magnetic moments for **a** $J = 0.4$ eV, **b** $J = 0.6$ eV , **c** $J = 0.8$ eV , and **d** $J = 1$ eV for different magnetic configurations vs different values of pressure, all at $U = 4$ eV, respectively.

[1] Baroni, S., Giannozzi, P. & Testa, A. Greens-function approach to linear response in solids*Phys. Rev. Lett.* **58**, 1861 (1987).
[2] Gonze, X. Perturbation expansion of variational principles at arbitrary orderPhys. Rev. A **52**, 1086 (1995).
[3] Gonze, X. Adiabatic density-functional perturbation theory*Phys. Rev. A* **52**, 1096 (1995).
[4] Chaput, L., Togo, A., Tanaka, I. & Hug, G. Phonon-phonon interactions in transition metals*Phys. Rev. B* **84**, 094302 (2011).
[5] Togo, A. Tanaka, & I. First principles phonon calculations in materials science*Scr. Mater.* **108**, 1 (2015).
[6] Mostofi, A. A. *et al.* Wannier90: A tool for obtaining maximally-localised wannier functions. *Comput. Phys. Commun.* **178**, 685-699 (2008).

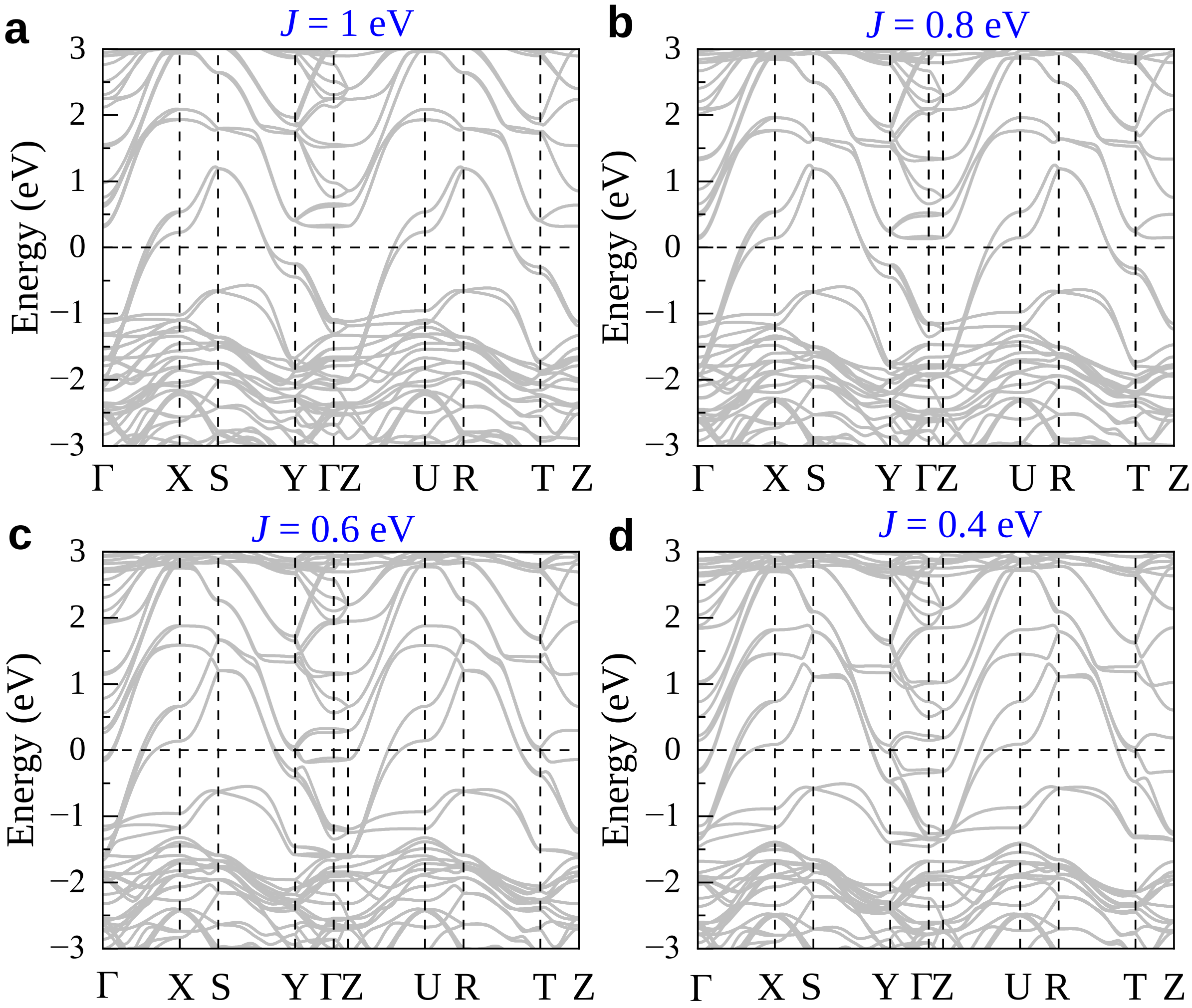

Supplementary Figure 9: **Band structures of the magnetic stripe state at 25 GPa.** The DFT calculated band structures for **a** $J = 1$ eV, **b** $J = 0.8$ eV , **c** $J = 0.6$ eV , and **d** $J = 0.4$ eV for the magnetic stripe state at 25 GPa, all at $U = 4$ eV, respectively.

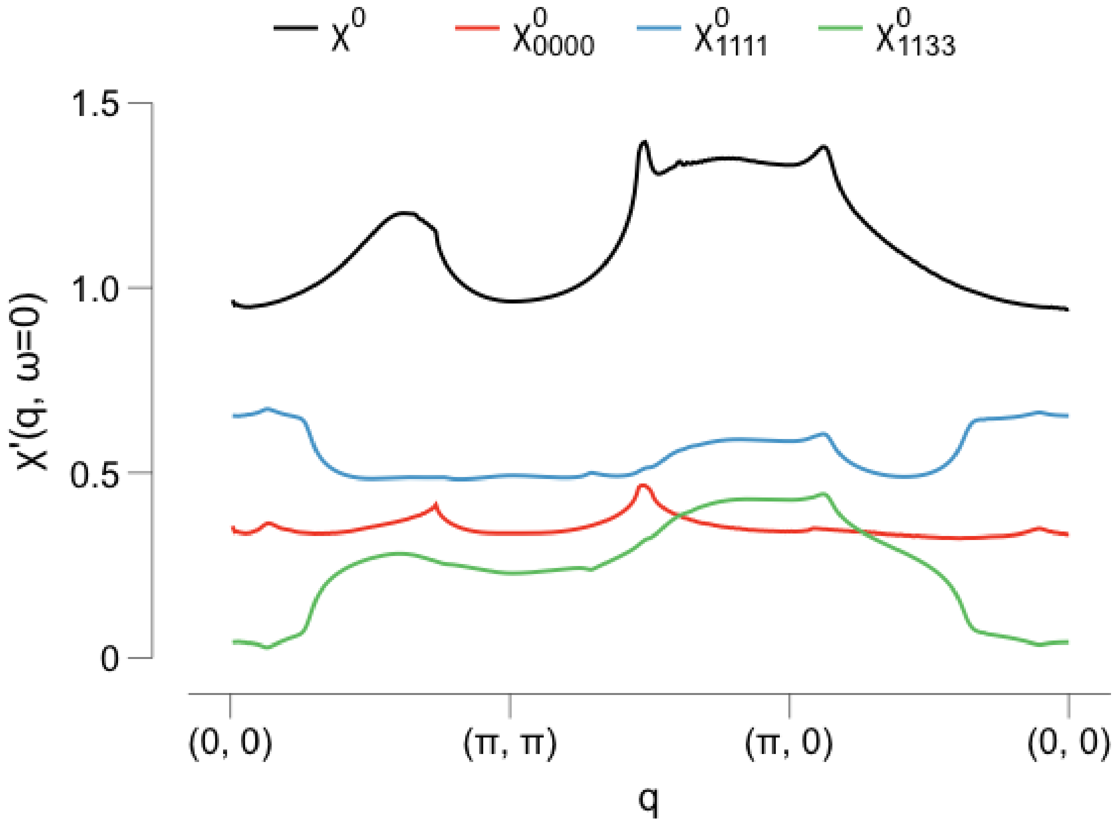

Supplementary Figure 10: **RPA bare susceptibility.** The RPA calculated static bare susceptibility $\chi'(\mathbf{q}, \omega = 0)$ vs. q_x, q_y for $q_z = \pi$ using the two-orbital bilayer TB model for three different pressures at 0 GPa. $\chi'(\mathbf{q}, \omega = 0)$ shows a broad peak centered approximately at $\mathbf{q} = (\pi, 0)$ (and symmetry related wavevectors). Here we used $U = 0.8$, $U' = 0.4$, $J = J' = 0.2$ in units of eV. The orbitals are indexed as "0" for the $d_{x^2-y^2}$ orbital in layer1,"1" for the $d_{3z^2-r^2}$ orbital in layer1, "2" for the $d_{x^2-y^2}$ orbital in layer2, and "3" for the $d_{3z^2-r^2}$ orbital in layer2.

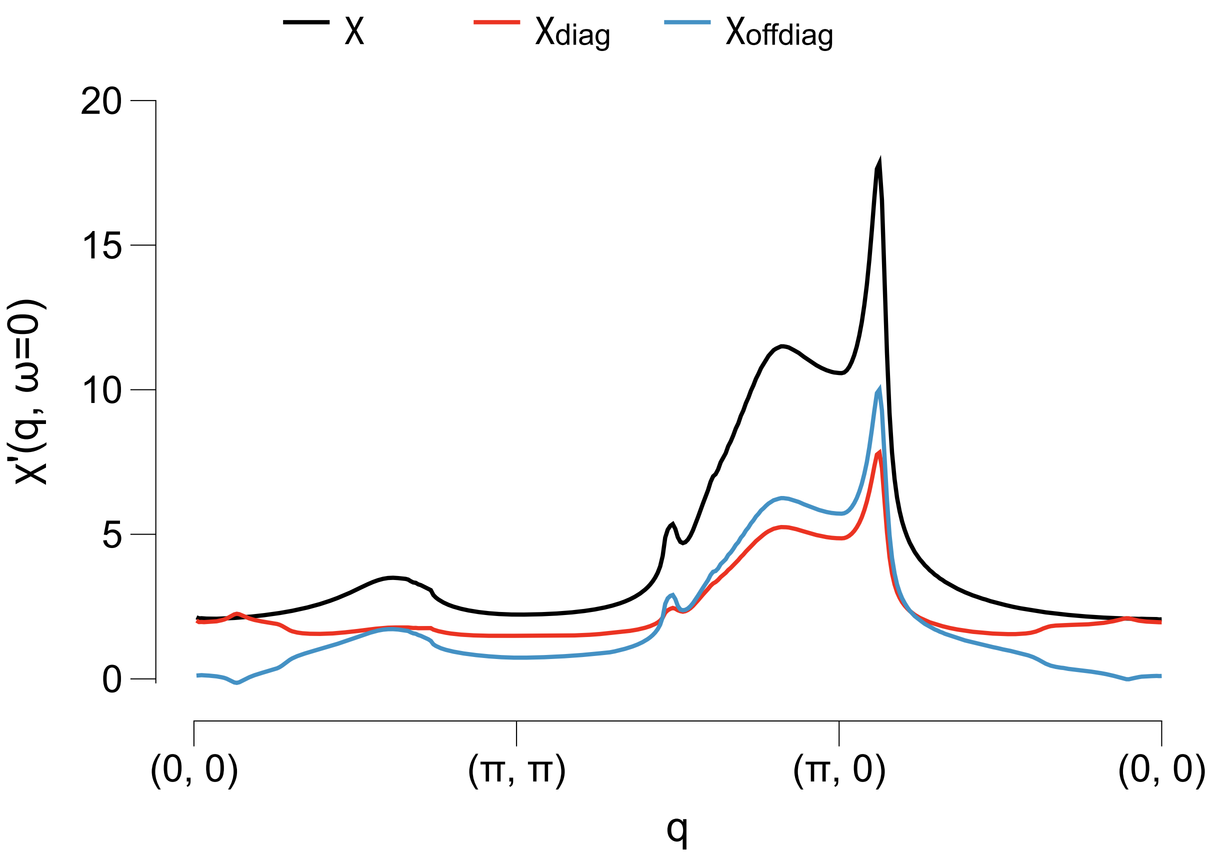

Supplementary Figure 11: **The detailed contributions of the RPA spin susceptibility.** The diagonal (red curve) and off-diagonal (blue curve) contributions to the RPA spin susceptibility at 0 GPa.

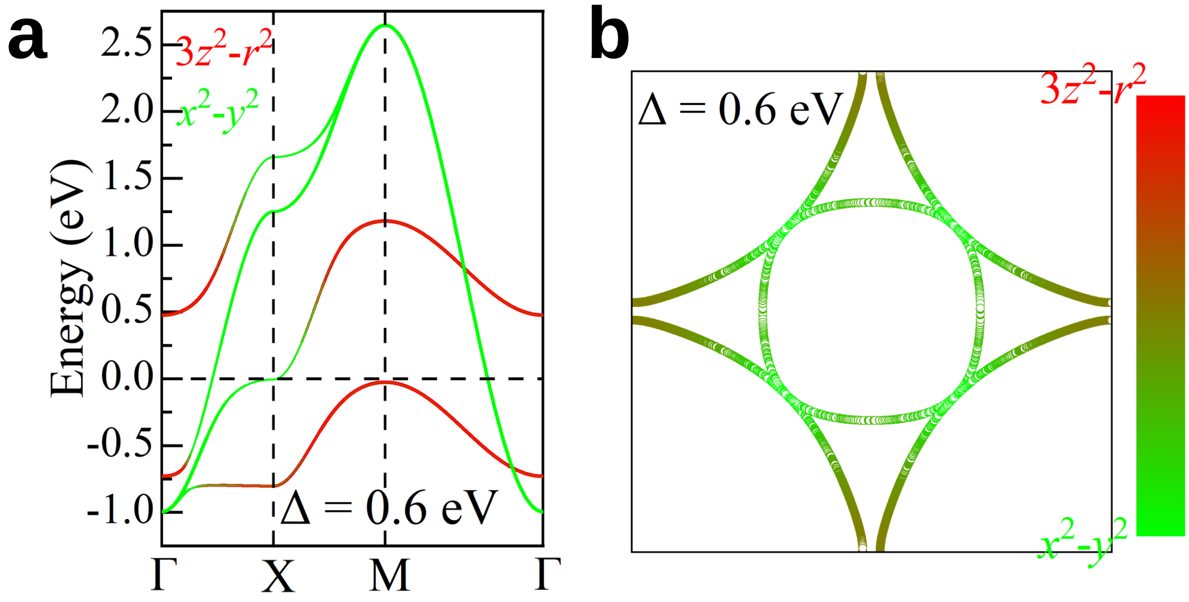

Supplementary Figure 12: **Band structure and Fermi surface with $\Delta = 0.6$ eV.** **a** Band structure and **b** Fermi surfaces for $\Delta = 0.6$ eV from TB calculations. Here, the hopping matrix for 0 GPa was used. Specifically, the hoppings used are: t_{11}^{x} = t_{11}^{y} = -0.088, t_{12}^{x} = 0.208, t_{12}^{y} = -0.208, t_{22}^{x} = t_{22}^{y} = -0.455, and t_{11}^{z} = -0.603. All the hoppings are in eV units. Note the absence of the pocket at (π, π) due to the increase in Δ, which also causes the $s^{\pm}$ pairing to stop dominating over d-wave, see main text.
